# Compact Surface Plasmon Resonance System with Au/Si Schottky Barrier

**DOI:** 10.3390/s18020399

**Published:** 2018-01-30

**Authors:** Takuya Tsukagoshi, Yuta Kuroda, Kentaro Noda, Nguyen Binh-Khiem, Tetsuo Kan, Isao Shimoyama

**Affiliations:** 1 Information and Robot Technology Research Initiative, The University of Tokyo, Tokyo 113-8656, Japan; tsukagoshi@leopard.t.u-tokyo.ac.jp; 2 Department of Mechano-Informatics, Graduate School of Information Science and Technology, The University of Tokyo, Tokyo 113-8656, Japan; kuroda@leopard.t.u-tokyo.ac.jp (Y.K.); noda@leopard.t.u-tokyo.ac.jp (K.N.); khiem@leopard.t.u-tokyo.ac.jp (N.B.-K.); 3 Graduate School of Informatics and Engineering, The University of Electro-Communications, Tokyo 182-8585, Japan; tetsuokan@uec.ac.jp

**Keywords:** surface plasmon resonance (SPR), Schottky barrier, diffraction grating, chemical sensor

## Abstract

Ethanol concentration was quantified by the use of a compact surface plasmon resonance (SPR) system, which electrically detects hot electrons via a Schottky barrier. Although it is well known that SPR can be used as bio/chemical sensors, implementation is not necessarily practical, due to the size and cost impediments associated with a system with variable wavelength or angle of incidence. However, scanning capability is not a prerequisite if the objective is to use SPR in a sensor. It is possible to build a small, inexpensive SPR sensor if the optics have no moving parts and a Schottky barrier is used for electrical current detection in place of a photodetector. This article reports on the design and performance of such a novel SPR sensor, and its application for quantifying ethanol concentration. As the concentration of ethanol is increased, the change in the angle dependence of the SPR current is observed. This change can be understood as a superposition of contributions of SPR coupled with the +3rd- and −3rd-order diffraction. Moreover, real-time monitoring of ethanol concentration was demonstrated using the proposed SPR system.

## 1. Introduction

Surface plasmon resonance (SPR) was first discovered in the 1950s [1]. However, it was not until the 1980s that the detailed physical properties of SPR, such as near-field optical phenomena, were well understood [2,3,4]. The potential application of SPR in the design of gas sensors [5] and biosensors [6] has dramatically accelerated the development of SPR-based technologies. Applications in physics, chemistry, biology, and material science are rapidly increasing and include the analysis of Langmuir-Blodgett film [7], self-assembled monolayers [8], detection of proteins and DNA [9], fluorescence spectroscopy [10], Raman scattering [11], and subwavelength imaging [12,13].

However, the optics for SPR-based systems are large and expensive, because reflection measurements are required, which involve the rotation of both a metallic film and a photodetector. Recently, the development of portable SPR-based sensors has been expected in clinical assays, environmental measurement, and other field studies [14]. Recently, an SPR setup with fixed optics was proposed [15]. The SPR system proposed in this paper is characterized by the electrical detection of SPR with a fixed angle of incidence. One of the key enabling technologies for compact and robust SPR-based sensor system development is the detection of SPR using an electric current, instead of optical reflection. It has been theoretically [16,17] and experimentally [18] demonstrated that SPR excites electrons in a metallic film that are not in thermal equilibrium (hot electrons). The phenomenon could be electrically detected using a rectification device such as a Schottky barrier. However, no reports have been published on chemical SPR-based sensors using electrical detection.

In this report, we propose an electrical detection SPR sensor system with no moving parts (solid state sensor), which is suitable for portable and robust sensing. The operating mechanism of the SPR sensor used in this paper is based on the theoretical framework depicted in [18], in which SPR was detected as a photocurrent. Our SPR sensor was designed to operate with the angle of incidence fixed to normal incidence when the sensor is dipped in water. As the concentration (refractive index) of the solution changes, the condition for exciting SPR is not satisfied, resulting in a decrease in the photocurrent. By making use of the dependence of the photocurrent on the refractive index, the concentration of the solution can be measured. Using the proposed sensor, we demonstrated that ethanol concentration could be quantified. Moreover, the use of a solid-state sensor facilitates real-time quantification, since angle and wavelength scanning is not required.

## 2. Materials and Methods

**Figure 1:**
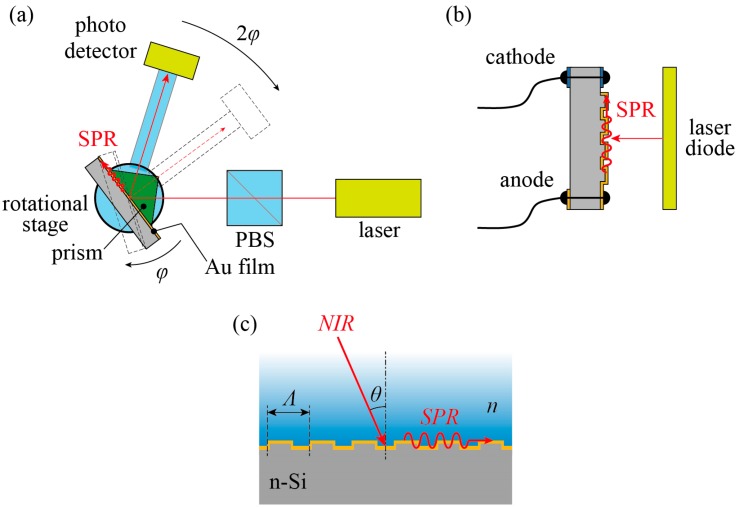
Schematic diagram of the experimental setups and detection principles. (**a**) Conventional optics to detect SPR using a double rotational stage; (**b**) Proposed chemical/biological sensor; (**c**) Mechanism of SPR excited on the grating by NIR laser illumination.

It is impossible to generate SPR on a flat metallic surface using an excitation source from the air side. Even if the surface is covered with a liquid (ethanol in this study), exciting SPR requires a prism-like container to transfer the tangential component of the wave vector. In conventional SPR sensors, the angle of incidence is varied and the reflectivity is measured with a photodetector, which is rotated around the irradiated point on the metallic film (Figure 1a). When the SPR device is rotated by *φ*, the photodetector has to be rotated by 2*φ*. Otherwise, both the laser source and the photodetector will be rotated, and the metallic film will remain fixed. Scanning the angle of incidence is not necessary to measure the concentration of solutions. The concentration can be quantified by changes in the photocurrent or reflectivity. The SPR sensor proposed in this study has no moving parts (Figure 1b). In our device, the pitch of the grating was designed to be 3.5 µm, so that the 3rd-order diffraction coupled to SPR for a wavelength of 1.55 µm [19]. The coupling of a near-infrared (NIR) laser source to SPR using a grating, has the advantage that the light from the laser diode is allowed to be normally incident onto the grating from the air side. This results in a more compact setup. In the case of a diffraction grating (Figure 1c), SPR is excited when

(1) $$\frac{\omega }{c}\sqrt{\varepsilon_{m}}\sin\theta +\frac{2n\pi }{\Lambda }=\frac{\omega }{c}\sqrt{\frac{\varepsilon_{m}\varepsilon \left(\omega \right)}{\varepsilon_{m}+\varepsilon \left(\omega \right)}}$$

is satisfied, where *ω* is the angular frequency of the laser, c is light speed, *θ* is the angle of incidence, *n* is diffraction order, *Λ* is the pitch of the grating, and $\varepsilon_{m}$ and ε(ω) are dielectric constants of ethanol and Au, respectively [19,20]. Therefore, SPR can be generated even in the case of normal incidence (θ=0). According to Equation (1), the 1st-, 2nd-, and 3rd-order diffraction of the normally incident light (*λ* = 1.55 µm) is coupled to SPR when the pitches of the grating are 1.17, 2.34, and 3.50 µm, respectively. In this experiment, 3rd-order diffraction was chosen to fabricate the diffraction grating with our photolithography processes. The referenced dielectric functions for gold [21] and water [22] were used to estimate the above grating pitch. A Schottky barrier was made at the interface between the n-Si substrate and Au film, so that hot electrons excited in the Au-film could be detected as a current [16,17]. Theoretical analysis of the SPR phenomenon suggest that the angle of incidence, for which the electric current is a maximum, shifts as the ethanol concentration is varied. Therefore, the ethanol concentration (refractive index) is quantified as a change of the current *I*, even if the angle of incidence is fixed to $\theta_{1}$. The electric current *I*_1_ changes to *I*_2_ and *I*_3_, as the refractive index *n*_1_ changes to *n*_2_ and *n*_3_, respectively.

**Figure 2:**
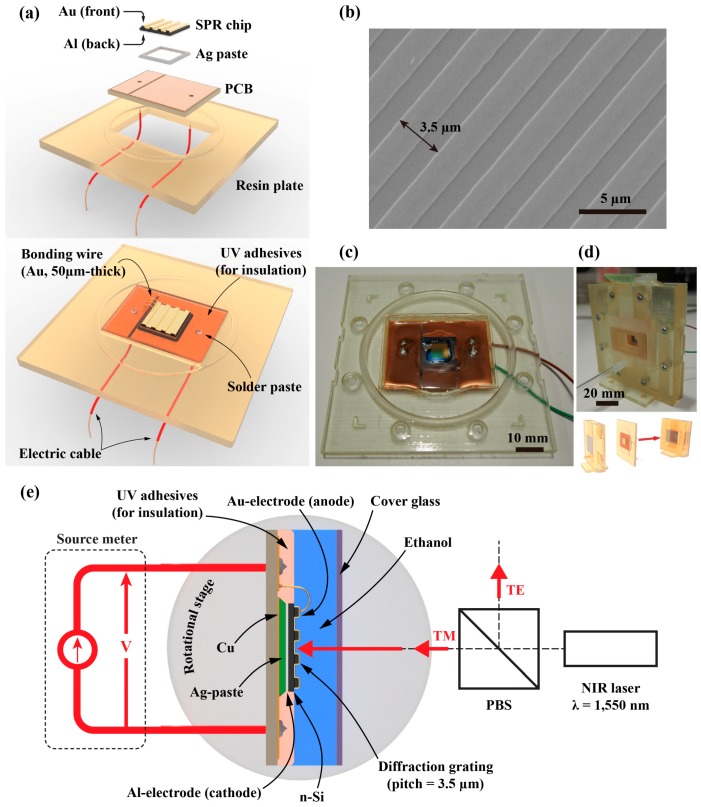
(**a**) Schematic of the proposed SPR sensor. The SPR chip is attached to the printed circuit board (PCB) with Ag paste, and electrically connected to the electric cable by solder paste. The liquid container to place the SPR chip into contact with the ethanol solution is also depicted; (**b**) Scanning electron microscope image of the diffraction grating coated with the Au film; (**c**) Photograph of the SPR sensor embedded on a PCB and a resin plate; (**d**) Photograph of the liquid container with the SPR sensor combined; (**e**) Optics for the experiments.

The diffraction grating was made on an n-Si wafer by the inductively coupled plasma-reactive ion etching (ICP-RIE) process. Then, a Au film of 100 nm thickness was deposited on the wafer by electron beam deposition (SPR chip). To ensure that the side walls of the grating were completely covered by the Au film, the electron beam deposition (JBS-Z0502EVC, JEOL Ltd., Tokyo, Japan) was obliquely performed. A 1-µm-thick Al layer was deposited on the backside of the Si wafer using thermal evaporation in a vacuum (SVC-700TM SG, Sanyu Electron, Tokyo, Japan). The SPR chip was attached to a printed circuit board (PCB) with Ag paste (Electroconductivity D-753A, Fujikura Kasei, Tokyo, Japan). The paste was hardened by annealing in a 100 °C environment for 30 min. The Au layer and the PCB were electrically connected using an ultrasonic wire bonder (Model 53xx, F&K Delvotec, Ottobrunn, Germany) (SPR circuit) (Figure 2a). This circuit was then attached to a resin plate. The grating had a pitch and depth of 3.5 µm and 130 nm, respectively (Figure 2b). The resin plate was combined with other components to form a chamber container (Figure 2c,d). Figure 2e indicates the optics that were used to measure SPR properties. The NIR laser source (Lambda mini, RGB Photonics) was introduced via an optical fiber and emitted to free space via a collimating lens. The NIR beam was then divided according to polarization using a polarized beam splitter (PBS), and only the TM component was allowed to be incident on the SPR chip. The power of the NIR laser at the source was 5 mW. A glass window in the container allowed the NIR laser beam to reach the surface of the grating. The electric current flow between the cathode (Al) and anode (Au) was detected by the source meter (2636B, KEITHLEY Instruments, Inc., Cleveland, OH, USA).

## 3. Results

**Figure 3:**
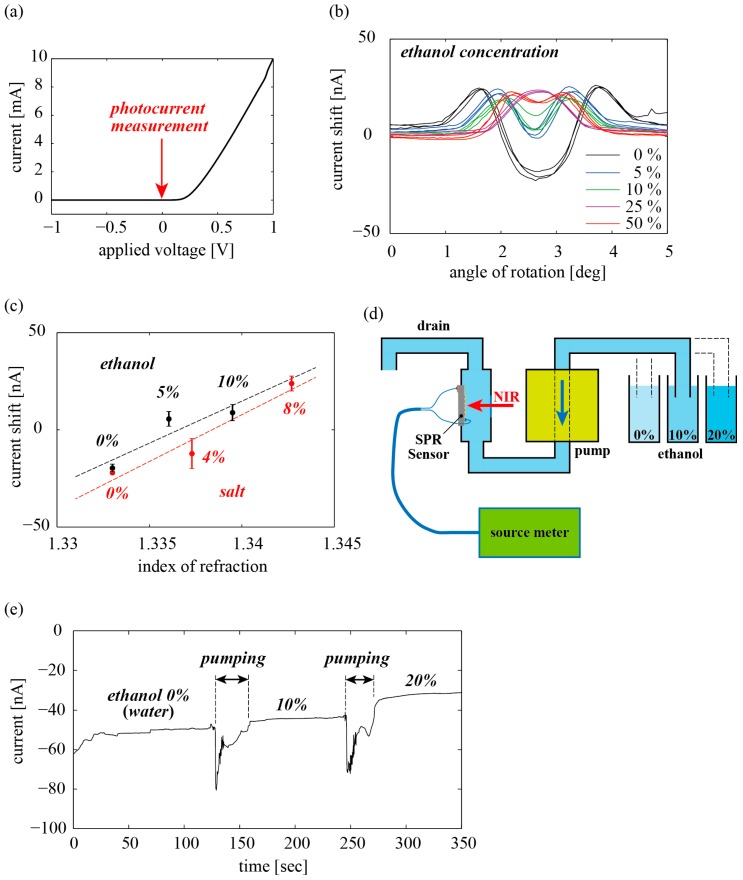
Results of the experiments; (**a**) IV-characteristics of the fabricated Schottky barrier; (**b**) Measured dependence of SPR current on angle of rotation for various ethanol concentrations; (**c**) Relation between refractive index of solution and SPR current for ethanol and salt; (**d**) Setup of concentration monitoring using a pump; (**e**) Result of the monitoring.

The electrical characteristics of the SPR chip were measured using a source meter. During this measurement process, the SPR chip was mounted on the PCB and resin plate, but not on the chamber container. The relationship between the applied voltage and generated current through the Schottky barrier is shown in Figure 3a. A well-defined rectification behavior was observed. The photon energy of the NIR laser (1.55 µm) is 0.80 eV, while the height of the Schottky barrier in our device was 0.78 eV [23]. Therefore, the hot electrons are effectively transduced to photocurrent. The forward resistance was 78 Ω over the forward voltage drop of ~0.2 V, and an electric current of 10 mA was attributed to the diffusion current carried by the bias voltage of 1 V. Next, the plate with the SPR chip and the PCB were integrated into the chamber container, and deionized water was subsequently added. In this proof-of-principle experiment, we employed a rotational stage to determine the SPR incident angles. A bias voltage of 0 V was applied to the SPR sensor during the measurement of the photocurrent, so that the measured current was on the order of nanoamperes. The measured current was generated by the photon energy of the incident light, instead of the diffusion current. After the laser was turned on, the stage was rotated, and the recorded current readings are shown in Figure 3b. The horizontal axis includes an error resulting from misalignment. From the obtained results, it was found that a rotation angle of 2.7° corresponds to normal incidence. The deionized water was replaced with 5%, 10%, 25%, and 50% ethanol (the original concentration being 99.5%), and the same measurements were repeated (three times for each concentration). The results of the photocurrent can be understood as a superposition of SPR coming from both the +3rd- and −3rd-order diffraction. The angular dependence of the photocurrent includes a peak coming from total internal reflection and a dip coming from excitation of SPR [20]. In other words, as the ethanol concentration increases, the angle of incidence deviates from the conditions under which SPR is excited. The measured current for each concentration at an angle of incidence of 2.8° is plotted against the refractive index (Figure 3c). The same plot is also shown in the case of salt (NaCl) solution with concentrations of 0%, 4%, and 8%. The refractive index for each concentration was obtained from the literature [22,24]. The approximated plots for salt solutions slightly differ from that for ethanol, which is possibly caused by ion adsorption on the gold film driven by the photocurrent.

Finally, the real-time monitoring of ethanol concentration was demonstrated using a pump, as depicted in Figure 3d. At first, the chamber container was filled with deionized water. The pump gradually replaced the water with 10% ethanol, two minutes after the photocurrent measurement was intiated. Once the replacement was completed, the pump was turned off. This was done to eliminate the effect of the pump noise on the measured data from the source meter. The 10% ethanol was replaced with 20% ethanol in the same manner, and the photocurrent during the replacement procedure is shown in Figure 3e.

## 4. Discussion

In this paper, a solid-state chemical sensor that electrically detects SPR with no moving parts has been proposed, designed, and fabricated. In Figure 3b, the current level changes according to the concentration of ethanol, even when the angle of incidence is fixed at 2.7°. A rotational stage was used to tune the SPR angle of the device. This tuning procedure will be effectively eliminated with appropriate design changes. Even if the tuning procedure is required, it could be replaced by wavelength tuning using inexpensive, single-wavelength laser diodes. This is feasible because the tuning range is small. As such, adjustment of the bias current or temperature of the diode is sufficient to accomplish this task. Using the proposed approach, the quantitative evaluation of temporal changes in the concentration of ethanol was achieved using a fixed wavelength and angle of incidence (Figure 3d,e). The baseline of the current in Figure 3e seemed to gradually increase, which was possibly due to temperature elevation by the laser irradiation or remnants of old solutions. When the temperature of the Schottky junction increases, the quantum efficiency of the photoelectric conversion decreases. This issue can be resolved by adding a dummy Schottky barrier nearby that has the same electrical characteristics with the sensor element. The effect of the thermal drift can be eliminated by making a differential measurement between the dummy Schottky element and the sensor element. The photocurrent varied with the angle, depending on the concentration, which is understood as contributions of the +3rd- and the −3rd-order diffraction. The photocurrent caused by SPR is estimated to be 25 nA. Taking the input laser power of 5 mW into consideration, we determined the responsivity of the SPR sensor to be 5 nA/mW. We used 3rd-order diffraction from the grating with 3.50 µm-pitch to excite SPR due to limitation of a photolithography equipment in our lab. However, the responsivity could be improved by modifying the structural arrangement of the diffraction grating. For example, Sobhani et al. [17] engineered the plasmonic structure such that SPR can be excited at the metal/Si interface and obtained a responsivity as high as hundreds nA/mW. It is noteworthy that in the diagram illustrating the relationship between the refractive index and the photocurrent, the measured values for ethanol and salt are in close agreement. Therefore, the response of the sensor represents a photocurrent due to SPR, which is influenced by the refractive index (electric permittivity).

## References

[1] Ritchie R.H. (1957). Plasma losses by fast electrons in thin films. Phys. Rev..

[2] Fischer U.C., Pohl D.W. (1989). Observation of single-particle plasmons by near-field optical microscopy. Phys. Rev. Lett..

[3] Berthold K., Höpfel R.A., Gornik E. (1985). Surface plasmon polariton enhanced photoconductivity of tunnel junctions in the visible. Appl. Phys. Lett..

[4] Brueck S.R.J., Diadiuk V., Jones T., Lenth W. (1985). Enhanced quantum efficiency internal photoemission detectors by grating coupling to surface plasma waves. Appl. Phys. Lett..

[5] Nylander C., Liedberg B., Lind T. (1982). Gas detection by means of surface plasmon resonance. Sens. Actuators.

[6] Liedberg B., Nylander C., Lunström I. (1983). Suface plasmon resonance for gas detection and biosensing. Sens. Actuators.

[7] Rothenhäusler B., Knoll W. (1988). Surface-plasmon microscopy. Nature.

[8] Tamada K., Ishida T., Knoll W., Fukushima H., Colorado R., Graupe M., Shmakova O.E., Lee T.R. (2001). Molecular packing of semifluorinated alkanethiol self-assembled monolayers on gold: Influence of alkyl spacer length. Langmuir.

[9] Homola J. (2008). Surface plasmon resonans sensors for detection of chemical and biological species. Chem. Rev..

[10] Liebermann T., Knoll W., Sluka P., Herrmann R. (2000). Complement hybridization from solution to surface-attached probe-oligonucleotides observed by surface-plasmon-field-enhanced fluorescence spectroscopy. Colloids Surf. A.

[11] Xu H., Bjerneld E.J., Käll M., Börjesson L. (1999). Spectroscopy of single hemoglobin molecules by surface enhanced raman scattering. Phys. Rev. Lett..

[12] Pendry J.B. (2000). Negative refraction makes a perfect lens. Phys. Rev. Lett..

[13] Fang N., Lee H., Sun C., Zhang X. (2005). Sub-diffraction-limited optical imaging with a silver superlens. Science.

[14] Mauriz E., Calle A., Montoya A., Lechuga L.M. (2006). Determination of environmental organic pollutants with a portable optical immunosensor. Talanta.

[15] Connolly P.W.R., Kaplan A. (2016). Demonstrating the angular, wavelength and polarization dependence of surface plasmon resonance on thin gold films—An undergraduate experiment. Am. J. Phys..

[16] Clavero C. (2014). Plasmon-induced hot-electron generation at nanoparticle/metal-oxide interfaces for photovoltaic and photocatalytic devices. Nat. Photonics.

[17] Sobhani A., Knight M.W., Wang Y., Zheng B., King N.S., Brown L.V., Fang Z., Nordlander P., Halas N.J. (2013). Narrowband photodetection in the near-infrared with a plasmon-induced hot electron device. Nat. Commun..

[18] Chen W., Kan T., Ajiki Y., Matsumoto K., Shimoyama I. (2016). Nir spectrometer using a schottky photodetector enhanced by grating-based spr. Opt. Express.

[19] Kan T., Matsumoto K., Shimoyama I. (2010). Tunable gold-coated polymer gratings for surface plasmon resonance coupling and scanning. J. Micromech. Microeng..

[20] Raether H. (1988). Surface Plasmons on Smooth and Rough Surfaces and on Grating.

[21] Johnson P.B., Christy R.W. (1972). Optical constants of the noble metals. Phys. Rev. B.

[22] bin Mat Yunus W.M., bin Abdul Rahman A. (1988). Refractive index of solutions at high concentrations. Appl. Opt..

[23] Cheung S.K., Cheung N.W. (1986). Extraction of schottky diode parameters from forward current-voltage characteristics. Appl. Phys. Lett..

[24] Nowakowska J. (1939). The Refractive Indices of Ethyl Alcohol and Water Mixtures. Master’s Thesis.

